# Coronary atherosclerosis and chemotherapy: From bench to bedside

**DOI:** 10.3389/fcvm.2023.1118002

**Published:** 2023-01-19

**Authors:** Fanghui Zhou, Xinxin Zhu, Yao Liu, Yue Sun, Ying Zhang, Dechun Cheng, Wei Wang

**Affiliations:** ^1^Department of Hematology, The Second Affiliated Hospital of Harbin Medical University, Harbin, Heilongjiang, China; ^2^Department of Endocrinology and Metabolism, The Second Affiliated Hospital of Harbin Medical University, Harbin, Heilongjiang, China; ^3^Department of Blood and Endocrinology, The 962nd Hospital of the PLA Joint Logistic Support Force, Harbin, Heilongjiang, China; ^4^Key Laboratory of Preservation of Human Genetic Resources and Disease Control in China (Harbin Medical University), Ministry of Education, Harbin, China; ^5^Qiqihar First Hospital, Qiqihar, China

**Keywords:** coronary atherosclerosis, chemotherapy, anthracycline, proteasome inhibitors, cardiovascular disease

## Abstract

Cardiovascular disease, particularly coronary artery disease, is the leading cause of death in humans worldwide. Coronary heart disease caused by chemotherapy affects the prognosis and survival of patients with tumors. The most effective chemotherapeutic drugs for cancer include proteasome inhibitors, tyrosine kinase inhibitors, immune checkpoint inhibitors, 5-fluorouracil, and anthracyclines. Animal models and clinical trials have consistently shown that chemotherapy is closely associated with coronary events and can cause serious adverse cardiovascular events. Adverse cardiovascular events after chemotherapy can affect the clinical outcome, treatment, and prognosis of patients with tumors. In recent years, with the development of new chemotherapeutic drugs, new discoveries have been made about the effects of drugs used for chemotherapy on cardiovascular disease and its related mechanisms, such as inflammation. This review article summarizes the effects of chemotherapeutic drugs on coronary artery disease and its related mechanisms to guide efforts in reducing cardiovascular adverse events during tumor chemotherapy, preventing the development of coronary heart disease, and designing new prevention and treatment strategies for cardiotoxicity caused by clinical tumor chemotherapy.

## 1. Introduction

The efficacy and safety of drugs are always a public concern. Optimal drugs, including those used for chemotherapy, are expected to have good therapeutic effects and minimal adverse effects. With significant advances in the treatment of cancer, the survival rates of patients have increased and their outcomes have improved. However, many chemotherapeutic drugs have cardiotoxic effects that can lead to important health problems such as cardiovascular disease. Commonly used chemotherapeutic drugs include proteasome inhibitors, tyrosine kinase inhibitors (TKIs), immune checkpoint inhibitors (ICIs), and anthracyclines. These drugs are among the most effective treatments for tumors and are widely used in clinical practice with proven efficacy. However, their use is often limited by their toxicity to the cardiovascular system. Notably, some anticancer drugs can cause coronary atherosclerotic heart disease. Atherosclerotic lesions of the coronary arteries can lead to vascular cavity stenosis or obstruction, resulting in myocardial ischemia, hypoxia, or necrosis. Coronary atherosclerotic heart disease is associated with high incidence and mortality rates. Studies have shown that pediatric cancer survivors who have undergone chemotherapy have considerably high rates of stroke and coronary artery disease and an 8.2 times higher cardiac mortality than expected (1). One study found that nearly 50% of pediatric cancer patients have been exposed to anthracyclines (2). Other studies have suggested that as many as one in eight childhood cancer survivors with anthracycline exposure will experience life-threatening cardiovascular events 30 years after treatment (3), whereas adult patients treated with anthracyclines will develop cardiotoxicity within 5 years. Several animal and cell experiments have demonstrated that programmed cell death protein 1 (PD-1), programmed death ligand 1 (PDL-1), and cytotoxic T-lymphocyte-associated protein 4 (CTLA-4) are key negative regulators of atherosclerosis (4–6). Clinical analyses have shown that, compared with women, men are at a higher risk of coronary artery disease and myocardial infarction associated with adverse immune reactions due to ICIs (7). Other studies have shown that the overall case fatality rate is as high as 36% (8). TKIs can also cause arterial thrombosis, which can lead to acute coronary syndrome and myocardial infarction in severe cases. The incidence of clinical arterial occlusion in patients with tumors treated with imatinib is 1.2–33.3%, and the percentage of vascular adverse events during the administration of nilotinib in patients with chronic myelogenous leukemia (CML) is 1–29% (9). Proteasome inhibitors are among the most effective drugs for the treatment of multiple myeloma (MM). The incidence of cardiovascular adverse events associated with carfilzomib use is considerably higher than that associated with bortezomib use (10). Several laboratory studies have also demonstrated that chronic proteasome inhibition is associated with increased coronary oxidative stress and early atherosclerosis formation (11). In addition, the mechanism of 5-fluorouracil (5-FU)-induced cardiotoxicity involves thrombosis secondary to vascular endothelial injury, ischemia secondary to coronary vasospasm, direct toxicity to the myocardium, and primary thrombosis (12).

This review article provides an overview of the relationship between some chemotherapeutic agents and coronary atherosclerosis and summarizes the effects of some chemotherapeutic drugs on coronary events. In addition, the mechanism of the effect of chemotherapeutic agents on the formation of coronary atherosclerotic plaques and the management of patients are explored to lay a foundation for the safety of tumor therapy and, to a certain extent, elucidate the occurrence of cardiovascular adverse events during tumor treatment. The purpose of this review is to guide efforts in reducing the occurrence of malignant cardiac events caused by chemotherapeutic drugs, prolonging the survival rates, and improving the prognosis of patients.

## 2. Chemotherapeutic drugs

### 2.1. Anthracyclines

Anthracyclines, including Adriamycin (doxorubicin), Adriamycin liposomes, and daunorubicin, are widely used in the treatment of various malignant tumors, and clinical trials of these drugs began in the 1960s. The cardiotoxic effects of anthracyclines were first identified by Machado et al. (13) in a clinical trial involving 94 patients (68 children and 26 adults) with various tumor types. This trial summarized the clinical experience with daunorubicin (13). Ewer et al. (14) reported the cardiovascular adverse effects of anthracyclines in a multicenter retrospective analysis of 4,018 patients. After treatment, electrocardiographic changes associated with Adriamycin were observed in 456 patients. The most common electrocardiographic changes were ST–T wave alterations (176 cases), and 14 patients had myocardial infarction (14). Early studies on the cardiotoxicity of anthracyclines mostly focused on heart failure and myocarditis, whereas few studies focused on coronary atherosclerosis. However, we found numerous studies reporting data on the damaging effects of anthracyclines on the cardiovascular system. Chow et al. (15) conducted a preliminary study on the endothelial cytotoxicity of anthracyclines in pediatric cancer patients. They evaluated 14 cancer patients aged 4–12 years who had completed chemotherapy with an at least 300 mg/m^2^ dose of anthracyclines 2–60 months before the study. Established measures of endothelial diastolic function, such as brachial artery reactivity, were significantly different between patients who received at least 300 mg/m^2^ anthracyclines and control patients (15). Brachial artery reactivity can be used to evaluate microvascular and ductal endothelial functions. During a brachial artery reactivity test, blood flow to the brachial artery is blocked and subsequently released to stimulate an endothelium-dependent vasodilation response, which predicts coronary atherosclerosis (16). Anthracyclines have been found to cause impaired endothelial function, which is an early event in the development of atherosclerosis, and the relationship between endothelial dysfunction and coronary atherosclerotic lesions is well established (17). Duncan et al. (18) also demonstrated that anthracyclines can increase the probability of dyslipidemia. They examined the incidence and mortality of advanced cardiovascular disease in 661 pediatric patients who had undergone hematologic malignancy transplantation and showed that those who received anthracycline chemotherapy before transplantation were more likely to be diagnosed with dyslipidemia than those who did not receive anthracycline treatment. They observed that for every 10 mg/m^2^ increase in the cumulative dose of anthracyclines, the risk of dyslipidemia increased by 1% (18). As dyslipidemia is the most important risk factor for arteriosclerosis (19), this adds to evidence suggesting that anthracyclines may cause cardiovascular disease and coronary atherosclerotic heart disease. Kalábová et al. (20) evaluated 36 patients with breast cancer before and after anthracycline chemotherapy. Their data showed that anthracycline chemotherapy was associated with increased levels of laboratory risk factors and the progression of atherosclerosis in patients with breast cancer (20). In another study, 243 patients with newly diagnosed breast cancer received anthracycline and other drugs (Table 1). After chemotherapy, the incidence of hyperlipidemia, level of blood glucose, and prevalence of hyperuricemia was significantly increased, and five patients (2.1%) were diagnosed with coronary artery disease (21). A recent clinical study showed significant increases in the mean carotid intima–media thickness and plaque score in 32 patients who received R-CHOP chemotherapy (rituximab, cyclophosphamide, doxorubicin, vincristine, and prednisolone) for malignant B-cell lymphoma. R-CHOP, an anthracycline-containing chemotherapy, has also been shown to promote atherosclerosis (22).

**TABLE 1:** Overview of clinical studies on cardiovascular toxicity of anthracyclines.

Drugs	References	Patients	Adverse cardiovascular events	Probability
Adriamycin	Ewer et al. (14)	4,018 patients with tumors	ST–T wave changes	4.4%
			Myocardial infarction	0.35%
Adriamycin	Chow et al. (15)	14 children with cancer	Impaired endothelial function	
	Duncan et al. (18)	661 children with hematologic tumors	Risk of dyslipidemia	
Adriamycin	Kalábová et al. (20)	36 patients with breast cancer	Arteriosclerosis	
	Dong et al. (21)	243 patients with newly diagnosed breast cancer	Hyperlipidemia	2.1% (5 cases)
			Elevated blood sugar level	
			High uric acid level	
			Coronary artery disease	
Doxorubicin	Shimizu et al. (22)	32 patients with malignant B-cell lymphoma	Arteriosclerosis	

### 2.2. Immune checkpoint inhibitors

ICIs, such as PD-1, PDL-1, and CTLA-4, have been used to treat various types of cancer. In recent years, diverse studies on the relationship between immunosuppressants and coronary atherosclerosis have been published. In a single-center clinical study, Drobni et al. (23) preliminarily evaluated whether ICI exposure was associated with atherosclerotic cardiovascular events in 2842 patients and 2842 controls. Their results showed that patients treated with ICIs had a three times increased risk of cardiovascular events and a more than three times increased rate of progression of total aortic plaque volume (23). A meta-analysis by Hu et al. (24) provided data on the severe immune-related adverse effects of PD-1 and PDL-1 inhibitors in patients with non-small cell lung cancer, demonstrating a 1.0% incidence of myocardial infarction and a 0.7% incidence of treatment-related death (24). In another single-center retrospective study that investigated 1,215 patients, those who developed myocardial infarction within 6 months after ICI treatment accounted for 1% of the total patients (25). Mahmood et al. (26) conducted a multicenter retrospective and prospective analysis of cardiovascular outcomes in patients with myocarditis after ICI treatment. They compared 35 patients with ICI-associated myocarditis and 105 randomly selected patients without myocarditis after ICI treatment. Their results showed that 46% of patients with myocarditis after immunotherapy experienced serious cardiovascular adverse events (26). Although relatively few clinical data are available on the influence of ICIs on coronary artery disease, the occurrence of acute vascular events in patients treated with ICIs has been shown to reduce the overall survival rate. In a study that analyzed 30 cases of ICI-related cardiotoxicity, 27% of patients died of cardiovascular complications (27). These results indicate that atherosclerotic cardiovascular events induced by ICI treatment affect the prognosis and quality of life of patients treated with ICIs. Although the incidence of cardiotoxicity associated with ICIs was not as high as that associated with anthracyclines, the late cardiovascular outcomes after ICI treatment were not optimistic and the rates of cardiovascular complications and mortality were not low. Therefore, the effects of ICIs on patient outcomes should not be ignored because of the low incidence of cardiotoxicity (Table 2). With the increasing application of ICIs, related cardiovascular risk management is also an urgent concern, highlighting the need for the development and clinical application of new immunotherapies with a focus on atherosclerosis and cardiovascular safety issues.

**TABLE 2:** Progress of clinical research on cardiovascular toxicity of immune checkpoint inhibitors.

Drugs	References	Patients	Adverse cardiovascular events	Probability
	Drobni et al. (23)	2,842 patients with ICI exposure	Increased aortic plaque volume	
	Mahmood et al. (26)	Patients after ICI treatment	Major adverse cardiovascular events	46%
PD-1 PDL-1	Hu et al. (24)	Patients with non-small cell carcinoma	Myocardial infarction	1%
	Duncan et al. (18)	66 children with hematologic malignancy	Coronary artery disease	0.2%
	Bar et al. (25)	1,215 patients after ICI treatment	Myocardial infarction	1%
	Escudier et al. (27)	30 patients with ICI-induced myocarditis	Mortality from cardiovascular complications	27%

ICI, immune checkpoint inhibitor; PD-1, programmed cell death protein 1; PDL-1, programmed cell death ligand 1.

### 2.3. Tyrosine kinase inhibitors

TKIs are a class of compounds that inhibit the activity of tyrosine kinases. These drugs are used to treat CML, non-small-cell lung cancer, renal cell carcinoma, and other cancers. Imatinib, a first-generation representative TKI, has transformed the treatment of CML; however, its safety has been a topic of discussion. The most common adverse reactions to imatinib include edema, gastrointestinal reactions, and cutaneous adverse reactions (28). The possible cardiotoxicity of imatinib was first reported by Grela-Wojewoda et al. (29). They examined 10 patients who developed severe heart failure during imatinib treatment; however, they found no direct relationship between imatinib and coronary atherosclerosis (29). Later studies reported that approximately 25% of patients respond well to the initial imatinib treatment but later develop resistance (30). Consequently, second-generation TKIs were developed to overcome imatinib resistance. However, nilotinib has been shown to accelerate atherosclerosis (31). Hochhaus et al. (32) evaluated the efficacy and safety of nilotinib in a phase 3 clinical trial in which 21 patients (7.5%) were treated with nilotinib 300 mg twice daily, nilotinib 400 mg twice daily, and imatinib. Nilotinib was associated with a higher incidence of coronary artery disease, which developed in 37 (13.4%) and 6 (2.1%) patients (32). Earlier Framingham studies clearly showed that patients with diabetes mellitus have a 2- to 4-fold increased risk of coronary atherosclerotic heart disease. This may also be related to the higher incidence of increased cholesterol and glucose levels, which increased the risk of coronary atherosclerosis, in the nilotinib group than in the imatinib group (33). Another study showed that 7.5% of patients treated with nilotinib developed acute coronary syndrome, among whom two patients had acute myocardial infarction and one patient died (34). In another single-center retrospective study that also demonstrated nilotinib-associated cardiovascular toxicity, Petrikova et al. (35) evaluated cardiovascular events in 82 patients with CML who received nilotinib or imatinib. In the nilotinib group, 14% of patients had one or more cardiovascular events, with myocardial infarction accounting for 4% of all events (35). The effects of sunitinib and sorafenib on tumor-associated endothelial cells may also lead to the activation of systemic coagulation in patients with cancer, making them more susceptible to thromboembolism. A meta-analysis showed that 122 of 9,387 patients experienced an arterial thromboembolic event (1.4%), and the incidence was 1.7% in the sorafenib group and 1.3% in the sunitinib group (36). In addition, as a new-generation TKI, ponatinib has shown a propensity to cause serious vascular adverse events in patients (31). The results from the phase 2 PACE trial of ponatinib conducted by Cortes et al. (37) showed that ventricular fibrillation (6%) and angina (5%) were among the serious cardiovascular adverse events reported in > 5% of patients with CML. In the general population, ventricular fibrillation, and angina were reported to occur in 4 and 3%, respectively (37). Interestingly, although TKIs have some cardiovascular adverse effects, imatinib has been reported to potentially have favorable metabolic and vascular effects (31) (Table 3). Imatinib has been shown to prevent and reverse type I diabetes and reduce diabetes-induced atherosclerosis in mice with prediabetes or newly diagnosed diabetes, possibly through the inhibition of platelet-derived growth factor receptor (PDGFR) (38, 39). PDGFR is a single-stranded transmembrane glycoprotein that is important in the PDGFR signal transduction pathway. The combination of PDGFR and its ligand can directly enhance the degradation of intercellular adhesion molecules and promote the invasion and metastasis of tumors. In a recent study on tubulointerstitial injury during renal congestion, the renal weight gain of male Sprague–Dawley rats treated with imatinib was significantly ameliorated and perivascular renal fibrosis was inhibited, as a result of PDGFR inhibition (40).

**TABLE 3:** Summary of clinical studies on cardiotoxicity of tyrosine kinase inhibitors.

Drugs	References	Patients	Adverse cardiovascular events	Probability
Nilotinib	Moslehi and Deininger (31)		Acceleration of arteriosclerosis	
Imatinib	Hochhaus et al. (32)	Imatinib 400 mg qd	Coronary artery disease	2.1%
Nilotinib		Nilotinib 300 mg bid		7.5%
		Nilotinib 400 mg bid		13.4%
Nilotinib	Kim et al. (60)	81 patients treated with nilotinib	Acute coronary syndrome	7.5%
			Acute myocardial infarction	2 cases
Nilotinib	Petrikova et al. (35)	82 patients with CML	Peripheral arterial obstructive disease Cardiomyopathy	10%
			Myocardial infarction	4%
Sunitinib	Choueiri et al. (36)	9,387 patients	Arterial thromboembolism	1.3%
Sorafenib				1.7%
Ponatinib	Cortes et al. (37)	Patients with CML	Ventricular fibrillation	6%
			Angina	5%

CML, chronic myelogenous leukemia; qd, once a day; bid, two times a day.

### 2.4. Proteasome inhibitors

Proteasome inhibitors, including bortezomib, carfilzomib, and ixazomib, are commonly used in the treatment of MM (41). The most common adverse effect of bortezomib is neurotoxicity (42). In a follow-up safety trial, Pancheri et al. (42) reported the cardiotoxicity of bortezomib in 69 patients. Eight of the 69 patients (11.6%) experienced serious cardiac adverse effects (42). Although controversy remains, the proteasome system is believed to play a role in the development and progression of atherosclerosis (43). Takamatsu et al. (44) reported the case of a 79-year-old woman with MM who had no history of heart disease and developed myocardial infarction on day 5 after treatment with bortezomib and dexamethasone. After stent treatment for coronary artery stenosis, the patient received another course of bortezomib and dexamethasone and developed angina again on day 5 after treatment. Bortezomib is a proteasome inhibitor that increases endothelial progenitor cell apoptosis and decreases endothelial nitric oxide synthase levels, resulting in coronary spasms (44). Data from phase 2 and 3 studies approved by US and EU regulatory authorities indicate that bortezomib has a 0.4–2.7% probability of causing coronary artery disease (45). In addition, in a prospective observational study, 17% of 30 patients with recurrent MM who received bortezomib experienced adverse cardiovascular events, whereas the incidence was 51% in patients who received carfilzomib (46). Carfilzomib is more likely than bortezomib to cause adverse cardiovascular reactions. A meta-analysis also showed that carfilzomib was associated with full-grade (incidence: 18.1%) and high-grade (incidence: 8.2%) cardiovascular adverse events (47). Another meta-analysis showed that among 5583 patients treated with carfilzomib, the incidence of full-grade hypertension, high-grade hypertension, and cardiovascular ischemia was 13.2, 5.3, and 4.6%, respectively (48) (Table 4). Jouni et al. (49) reported a case of cardiotoxicity induced by ixazomib, a novel oral proteasome inhibitor. A patient with MM with a history of paroxysmal ventricular fibrillation and no history of coronary artery disease was treated with lenalidomide, dexamethasone, cyclophosphamide, and prednisone. After the progression of MM, the patient was enrolled in a phase 2 clinical trial of ixazomib in combination with dexamethasone. After 2 months of treatment, labor-induced dyspnea and fatigue gradually developed. Cardiac evaluation at the end of the four-cycle trial showed that the patient had low levels of coronary atherosclerosis (49). Although proteasome inhibitors and anthracyclines have both been shown to have some cardiotoxicity, experimental data suggest that the combination of these two drug classes is not associated with an excessive risk of cardiotoxicity in the cardiomyocytes and heart tissues of young adult animals (50).

**TABLE 4:** Summary of clinical studies on cardiotoxicity of proteasome inhibitors.

Drugs	References	Patients	Adverse cardiovascular events	Probability
Bortezomib	Takamatsu et al. (44)	A 79-year-old woman with MM	Myocardial infarction	
Bortezomib	Laubach et al. (45)	3954 patients with MM	Coronary artery disease	0.4–2.7%
Bortezomib	Cornell et al. (46)	30 patients with recurrent MM	Adverse cardiovascular events	17%
Carfilzomib				51%
Carfilzomib	Waxman et al. (47)	2,594 patients with MM	Full-grade adverse cardiovascular events	18.1%
			High-grade adverse cardiovascular events	8.2%
Carfilzomib	Latif et al. (48)	5,583 patients	Cardiovascular ischemia	4.6%
			Full-grade hypertension	13.2%
			High-grade hypertension	5.3%
Ixazomib	Jouni et al. (49)	1 patient with MM	Coronary atherosclerosis	

MM: multiple myeloma.

### 2.5. 5-Fluorouracil

5-FU is a fluoride of the pyrimidine class that is commonly used in the treatment of malignancies such as colon, rectal, and gastric cancers. 5-FU can cause mild gastrointestinal symptoms, severe neutropenia, life-threatening cardiovascular events, and other adverse reactions. Early studies have identified the effects of 5-FU on the coronary arteries, and knowledge about its cardiovascular toxicity and adverse effects on the coronary arteries has improved with advances in treatments. The incidence of cardiovascular toxicity caused by 5-FU ranges from 2% to 18% (51, 52). Another retrospective analysis summarized the mortality risk from the cardiovascular adverse effects of 5-FU. In a study of 377 evaluable cancer patients treated with 5-FU alone or in combination with other drugs, 45% developed angina, 22% developed myocardial infarction, 8% died of 5-FU-related adverse effects, and 13% died of conditions associated with secondary exposure to 5-FU (53). Jensen and Sørensen (54) investigated the use of 5-FU in the treatment of vascular endothelial damage. They prospectively evaluated 106 patients with colorectal cancer before, during, and after chemotherapy with 5-FU. Their results showed that 5-FU can cause reversible systemic endothelial cell damage and the activation of a procoagulant state (54). In the early and late stages of arteriosclerosis, a variety of procoagulants and antithrombin proteases are immunohistochemically labeled at different locations on the arteriosclerotic vessel wall, whereas the procoagulant state of early plaques are enhanced (55). An earlier single-center study of 1083 cancer patients who underwent 5-FU chemotherapy showed cardiotoxicity in 17 patients, angina in 15 patients, and definitive myocardial infarction in 1 patient (56). Another prospective clinical trial of 367 patients treated with high-dose 5-FU showed 28 cardiac events induced by 5-FU, with 18 patients experiencing angina at the beginning of treatment and 21 patients developing unstable angina after the withdrawal of 5-FU (57). As studies in this period were limited by the available methods for the observation and detection of cardiac indicators, the influence of 5-FU on coronary atherosclerosis remains unclear. In a follow-up study, 4% of 427 patients who received continuous 5-FU infusion exhibited clinical symptoms and electrocardiographic abnormalities, 7 patients had myocardial infarction, 4 patients showed ischemic changes, and the incidence of cardiovascular adverse events was associated with the duration of 5-FU infusion (58). In addition, Wacker et al. (59) studied 102 patients treated with 5-FU. Although 19% of patients had reversible angina symptoms during treatment and six patients with severe angina underwent coronary angiography, none of the patients showed coronary artery disease on coronary angiography (59). Subsequently, Kim et al. (60) reported the case of an 83-year-old man with colon cancer who developed chest pain after 5-FU chemotherapy. The patient had no history of cardiovascular risk factors, and coronary angiography revealed significant atherosclerosis in the proximal left circumflex artery. Coronary spasm with fixed stenosis was considered in this case (60). In another case report showing that 5-FU can cause coronary atherosclerotic heart disease, a 59-year-old male patient with CML developed myocardial infarction after 5-FU treatment despite having no prior history of heart disease and no typical risk factors for coronary artery disease (Table 5). Emergency coronary angiography revealed a complete occlusion of the proximal descending branch of the left coronary artery (61).

**TABLE 5:** Summary of clinical studies on cardiotoxicity of 5-fluorouracil.

Drug	References	Patients	Adverse cardiovascular events	Probability
5-Fluorouracil	Saif et al. (53)	377 cancer patients	Angina	45%
			Myocardial infarction	22%
5-Fluorouracil	Ashish et al. (56)	1,083 cancer patients	Angina	15 cases
			Myocardial infarction	A case
5-Fluorouracil	Cherukuri et al. (57)	367 patients treated with high-dose 5-fluorouracil	Heart events	7.6%
			Angina	4.9%
5-Fluorouracil	Tsavaris et al. (58)	427 patients treated with continuous infusion of 5-fluorouracil	Abnormal ECG	4%
			Myocardial infarction	1.6%
			Ischemic changes in the heart	0.9%
5-Fluorouracil	Wacker et al. (59)	102 patients	Reversible angina pectoris	19%

ECG: electrocardiogram.

## 3. Preclinical studies

### 3.1. Anthracyclines

Although the mechanism of anthracycline cardiotoxicity has been widely discussed, no specific mechanism has been established to date. Anthracyclines influence endothelial cells, and endothelial cell dysfunction leads to reduced vasodilation and is believed to be an early event in atherosclerosis and other cardiovascular diseases. Impaired endothelium-dependent vasodilation is also observed in patients without risk factors for atherosclerosis (62, 63). Seara et al. (64) used an organ culture system to determine the damaging effects of doxorubicin on the vascular endothelium. Apoptosis and nuclear damage occurred in mesenteric artery endothelial cells treated with a high concentration (1 μM) of doxorubicin on day 3 of treatment, and endothelial cell detachment and stripping from the intima of the vascular wall were observed. Impairment of the endothelial diastolic function was observed on day 5. In addition, apoptotic changes in the smooth muscle layer were observed at this concentration of Adriamycin (64). Apoptosis of endothelial cells can lead to vascular thrombosis and endothelial stripping and may be a key step in the transition from stable endothelial plaques to plaque erosion and thrombosis (65). Meanwhile, anthracycline treatment is associated with progressive and irreversible damage to the coronary microcirculation. A recent animal study investigated changes in the coronary microcirculation status during and after anthracycline treatment. After cumulative injections of an anthracycline (0.45 mg/kg doxorubicin per injection into the coronary artery), the vascular function of isolated coronary arteries was evaluated using electromyography and the coronary flow reserve(CFR) was evaluated using cardiac magnetic resonance imaging. The results showed that low cumulative doses of doxorubicin caused sustained microcirculation damage, and histologic and electromyographic assessments confirmed structural damage in all arterial diameters, even in animals that received low cumulative doses. Conversely, high cumulative doses caused arteriolar damage and capillary bed changes. Low cumulative doses also caused this damage but did not affect the systolic function of the heart, which may further explain the increased incidence of cardiovascular events in patients treated with anthracyclines but had no observed problems in systolic function (66).

### 3.2. Immune checkpoint inhibitors

In recent years, the number of studies on the cardiovascular toxicity of ICIs has gradually increased. Although the complete pathogenesis of cardiovascular toxicity has not been clarified, ICIs have been found to play an important role in the development of atherosclerosis and the regulation of the coronary arteries. Bu et al. (67) studied the role of PD-1 pathway in regulating and promoting the formation of atherosclerotic lesions, and they found that after the use of blocking anti-PD-1 antibody, hypercholesterolemia Ldlr−/− mice had increased traumatic inflammation and a larger lesion than low-density lipoprotein receptor knockout mice (Ldlr−/−). Moreover, changes in the content of damaged T cells were observed in bone marrow chimeric Ldlr−/− mice lacking PDL-1 and PDL-2 hematopoietic cells when PD-1 was absent or blocked. This suggests that PD-1 plays an important role in downregulating the response of atherogenic T cells; therefore, blocking this molecule during cancer treatment may increase the risk of cardiovascular complications (67). This may be because ICIs immunize atherosclerotic T cells through the regulation of T-cell activation, leading to increased interferon-gamma and tumor necrosis factor (TNF)-alpha levels, thereby increasing the risk of coronary thrombosis (68). Poels et al. (69) also conducted a study on the effect of antibody-mediated CTLA-4 inhibition on experimental atherosclerosis in Ldlr−/− mice aged 6–8 weeks fed a 0.15% cholesterol diet for 6 weeks and treated with 200 μg CTLA-4 antibody every 2 weeks. Their results suggested that short-term antibody-mediated inhibition of CTLA-4 accelerates the progression of atherosclerosis by inducing T cells to drive an inflammatory response, resulting in the formation of plaques with larger necrotic cores and less collagen (69). Some experiments also found that T cells, the CD28–CD80/86 co-stimulation pathway, and the CTLA-4 co-inhibition pathway play a role in postintervention remodeling, and CTLA-4 + T cells show obvious intravascular infiltration (70). In another study that used a combination of anti-CTLA-4 and anti-PD-1 antibodies in hyperlipidemic mice, although the plaque size in the vessels of mice with coronary atherosclerosis was not affected, the plaque developed into a lymphoid-based inflammatory phenotype (71).

### 3.3. Tyrosine kinase inhibitors

TKIs cause high blood pressure, which is one of the risk factors for coronary atherosclerotic heart disease. According to previous studies (72), this is related to an increase in circulating endothelin-1 levels. Sunitinib can induce a reversible increase in blood pressure levels in humans and rats, which is related to the activation of the endothelin-1 system, the inhibition of the renin–angiotensin system, and systemic microvascular dysfunction. Drueckes et al. (73) reported that the perfect kinase targets of nilotinib are collagen receptor disk domain receptor 1 (DDR-1), DDR-2, BCR-Abl (Abl), PDGFRα/β, KIT, and colony-stimulating factor 1 receptor, and DDR-1 is associated with the formation of atherosclerotic plaques. *DDR-1* gene knockout is associated with increased plaque formation in a mouse model of atherosclerosis (74–76). KIT, also known as c-KIT, is a proto-oncogene that encodes a type III transmembrane receptor tyrosine kinase with tyrosine kinase activity. The receptor kinases KIT and PDGFR are involved in the regulation of various blood vessels and perivascular cells, and KIT is a major regulator of mast cell growth, survival, migration, and function. In addition, the production and release of histamine, heparin, and tissue plasminogen activator are also dependent on KIT (77). Thrombotic aggregates of mast cells have been found to surround blood vessels and provide many important repair molecules, including heparin, tissue plasminogen activator (a fibrinolytic precursor), and beta-trypsin (a fibrinolytic protease). Thus, recruitment and activation of mast cells may lead to local thrombolysis and the prevention of clotting (78). When drug-induced mast cell inactivation or depletion leads to the inhibition of the vascular repair system, thromboembolism and arterial stenosis events become more likely to occur (79). Therefore, nilotinib may affect mast cells and lead to cardiovascular events. Tie-2 is a receptor tyrosine kinase that is mainly expressed on the surface of endothelial cells and plays an important role in vascular stability, survival, and maturation (80). The fibroblast growth factor signaling pathway regulates tissue development, angiogenesis, and regeneration (81). Therefore, ponatinib inhibits the angiogenic receptor Tie-2 and all fibroblast growth factor receptor kinases, which may enhance vasotoxicity (82). In addition, vascular endothelial growth factor (VEGF) plays a complex role in balancing hemostasis and thrombosis, and changes in the function of this growth factor may lead to thrombosis or bleeding. TKIs can cause endothelial dysfunction, resulting in endothelial cell destruction or death of the barrier to the blood vessel wall. This ultimately leads to activation of platelets and clotting factors, resulting in thrombosis. Furthermore, this may explain how TKIs cause adverse cardiovascular events and myocardial infarction (83).

### 3.4. Proteasome inhibitors

Wu et al. reported (84) that the mechanism of the cardiotoxicity of proteasome inhibitors in myeloma is related to proteasome inhibition of IKKβ-dependent increased phosphorylation and downregulation of IκBα through the classical activation of nuclear factor-κB (85). Atherosclerosis is believed to be the result of increased endogenous oxidative stress. Proteasomes are responsible for the degradation of oxidative proteins, and their inhibition has been shown to induce oxidative stress *in vitro*. Herrmann et al. (11) suggested that the use of large amounts of proteasome inhibitors may impair the function and structure of the coronary arteries in normal animals and aggravate the vascular changes in hypercholesterolemic animals with early atherosclerosis. Previous studies have shown increased proteasome activity in the aorta of hypercholesterolemic rabbits (86); therefore, coronary artery studies were performed in hypercholesterolemic pigs. The results showed that substantial proteasome inhibition is associated with increased oxidative stress, impaired coronary endothelium-dependent vasodilation, and intimal thickening, exacerbating the vascular effects of traditional cardiovascular risk factors such as hypercholesterolemia (11). Notably, Ismawati et al. (87) found that proteasome inhibitors reduced early arteriosclerosis in low density lipoprotein receptor (LDLR) mice, this suggests that proteasome inhibitors inhibit the formation of early arteriosclerosis lesions (87). However, the effect of proteasome inhibitors on coronary atherosclerosis remains controversial. Most clinical studies indicate that proteasome inhibitors can cause coronary atherosclerosis and lead to cardiovascular problems, whereas some basic studies have come to the opposite conclusion. Therefore, further studies are required to elucidate this issue.

### 3.5. 5-Fluorouracil

Coronary artery spasm is the main manifestation of 5-FU-associated coronary artery disease. Coronary vasospasm may be associated with an endothelium-dependent mechanism (endothelial dysfunction) or an endothelium-independent mechanism (primary smooth muscle dysfunction) (88) and is an early manifestation of coronary atherosclerosis. Experimental evidence also supports the direct toxic effects of 5-FU on the coronary endothelium. Anaka and Abdel-Rahman (89) used electron microscopy to observe the endothelial cells of rabbit arterioles after 5-FU treatment. They studied the local and systemic effects of 5-FU on the endothelial cells of rabbits at 1, 3, 7, 14, and 30 days after 5-FU treatment. Severe cell damage associated with thrombosis was found (89). Clinical trial evidence also supports the direct toxic effects of 5-FU on the coronary endothelium and the hypercoagulable state, which together induce acute thrombotic events (54).

## 4. Cardiotoxicity prevention

Chemotherapeutic drugs have shown strong efficacy in the treatment of tumors, thereby considerably improving the survival rate of patients. However, the associated cardiotoxicity has limited the use of these drugs. Studies have shown that the long-term treatment outcomes of breast cancer survivors are largely dependent on atherosclerotic changes. Cardiovascular death is an important concern for older women with early breast cancer (90). In recent years, certain results have been achieved in the management and prevention of conditions induced by the cardiovascular toxicity of chemotherapeutic drugs, especially coronary atherosclerosis. Smith et al. (91) reported a 79% reduction in the risk of anthracycline cardiotoxicity with the use of the cardioprotective agent dexrazoxane. Dexrazoxane is an iron-chelating agent that is believed to reduce the cardiotoxic effects of doxorubicin by preventing the formation of free radicals (92). Conventional therapy is inadequate for ICI-induced cardiovascular toxicity. Large doses of corticosteroids should be administered if necessary. Immunosuppressive agents such as infliximab or mycophenolate should be considered in patients who do not immediately respond to high doses of corticosteroids (93). Aspirin and statins are currently considered prophylactic therapies for atherosclerosis associated with cancer treatment, and treatment with aspirin and statins should be considered in patients with a high risk of thrombosis and with many atherosclerosis risk factors. A meta-analysis found that although aspirin may reduce adverse cardiovascular events associated with chemotherapeutic drugs, it also increases the risk of bleeding (94). More attention should be paid to patients with secondary exposure to anticancer drugs. If long-term antithrombotic therapy is required, new anticoagulants may be considered to reduce the risk of thrombosis and bleeding (95). Statins reduce the risk of damage to the central blood vessels during anthracycline treatment (96). In another retrospective analysis of clinical data from 628 patients with newly diagnosed breast cancer treated with anthracyclines, the use of statins after anthracycline therapy was found to reduce the risk of cardiotoxicity in these patients (97). In addition to lowering cholesterol levels, statins can also reduce oxidative stress, inflammation, and the number of inflammatory cells in atherosclerotic plaques. Therefore, statins are effective in the treatment and prevention of coronary atherosclerosis (98). Beta-blockers block the action of endogenous catecholamines on beta-adrenergic receptors, thereby lowering blood pressure (99). Carvedilol is a non-selective β- and α-adrenergic receptor antagonist with a high antioxidant capacity. Its protective effects are attributed to its antioxidant capacity. Carvedilol effectively prevents endothelial dysfunction. Bosch et al. (100) conducted a study in which 90 patients with newly diagnosed acute leukemia or hematologic malignancies were randomly assigned to a combination treatment with enalapril and carvedilol or a control intervention. Their results showed that the death rate from cardiac events was 6.7% in the enalapril and carvedilol combined treatment group and 22% in the control group, showing that the combination treatment effectively reduced the death rate from cardiac events (100). Beta-blockers, angiotensin-converting enzyme inhibitors, angiotensin receptor blockers, aldosterone antagonists, diuretics, and other drugs can also treat and prevent the cardiotoxicity caused by proteasome inhibitors (84). Glucagon-like peptide-1 agonists may also play a role. Glucagon-like peptide-1 is a peptide secreted by intestinal endocrine cells and is mainly involved in stimulating glucose-dependent insulin secretion and insulin-stimulating signaling. Although it is mainly used to treat diabetes, glucagon-like peptide-1 is also involved in the regulation of inflammation and cardiovascular function (101). TNF is a pro-inflammatory cytokine mainly secreted by immune cells and is involved in inflammation, cell proliferation, apoptosis, and lipid metabolism. TNF drives inflammation and plaque formation in atherosclerosis; therefore, TNF inhibitors can be used to prevent coronary atherosclerosis (102). If the introduction of 5-FU is deemed the most effective option for a patient, then the risks and benefits of resuming treatment can be considered. Although the role of drugs in preventing 5-FU-induced coronary atherosclerosis has not been clearly defined, calcium channel blockers, long-acting nitrates, and beta-blockers have been used with varying degrees of success (103).

## 5. Conclusion

In the development of drugs for tumor treatment, from anthracyclines in the past to proteasome inhibitors in recent years, excellent therapeutic effects have been achieved, improving not only the survival rate but also the quality of life of patients. Although ample evidence suggests that treatment with oncologic drugs is associated with the development of coronary atherosclerosis, the mechanisms by which some drugs cause atherosclerosis have not been elucidated. This may be due to the insufficient number of clinical trial indicators and the lack of basic experimental research data. A comprehensive understanding of the relationship between the use of chemotherapeutic drugs and the formation of coronary atherosclerotic plaques can facilitate the development of relevant treatment strategies based on pathogenesis and timely preventive interventions. Therefore, further pathophysiologic experiments, clinical studies, and *in vivo* imaging studies need to be conducted in the future. We hope that the resulting additional knowledge will help reduce the occurrence of acute cardiovascular events and improve the survival rate of patients with cancer. The intersection between the treatment of cardiovascular diseases and the treatment of cancer also creates opportunities and challenges for the development of new drugs.

## Author contributions

FZ and XZ designed the framework and direction of the manuscript. YL and YS wrote the manuscript. YZ and DC collected literature and designed the tables. WW supervised the writing of the manuscript and revised the manuscript. All authors approved the final manuscript.
